# Tumor-infiltrating immune cells based TMEscore and related gene signature is associated with the survival of CRC patients and response to fluoropyrimidine-based chemotherapy

**DOI:** 10.3389/fonc.2022.953321

**Published:** 2022-08-30

**Authors:** Xian-Wen Guo, Si-Qi Li, Rong-E Lei, Zhen Ding, Bang-li Hu, Rong Lin

**Affiliations:** ^1^ Department of Gastroenterology, Union Hospital, Tongji Medical College, Huazhong University of Science and Technology, Wuhan, China; ^2^ Department of Gastroenterology, The People’s Hospital of Guangxi Zhuang Autonomous Region, Nanning, China; ^3^ Department of Research, Guangxi Medical University Cancer Hospital, Nanning, China; ^4^ Department of Gastroenterology, The First Affiliated Hospital of Guangxi Medical University, Nanning, China

**Keywords:** colorectal cancer, tumor-infiltrating immune cells, TMEscore, chemotherapy, machine learning, gene signature

## Abstract

**Background:**

Tumor-infiltrating immune cells (TIICs) are associated with chemotherapy response. This study aimed to explore the prognostic value of a TIIC-related tumor microenvironment score (TMEscore) in patients with colorectal cancer (CRC) who underwent chemotherapy and construct a TMEscore-related gene signature to determine its predictive value.

**Methods:**

Gene profiles of patients who underwent fluoropyrimidine-based chemotherapy were collected, and their TIIC fractions were calculated and clustered. Differentially expressed genes (DEGs) between clusters were used to calculate the TMEscore. The association between the TMEscore, chemotherapy response, and survival rate was analyzed. Machine learning methods were used to identify key TMEscore-related genes, and a gene signature was constructed to verify the predictive value.

**Results:**

Two clusters based on the TIIC fraction were identified, and the TMEscore was calculated based on the DEGs of the two clusters. The TMEscore was higher in patients who responded to chemotherapy than in those who did not, and was associated with the survival rate of patients who underwent chemotherapy. Three machine learning methods, support vector machine (SVM), decision tree (DT), and Extreme Gradient Boosting (XGBoost), identified three TMEscore-related genes (ADH1C, SLC26A2, and NANS) associated with the response to chemotherapy. A TMEscore-related gene signature was constructed, and three external cohorts validated that the gene signature could predict the response to chemotherapy. Five datasets and clinical samples showed that the expression of the three TMEscore-related genes was increased in tumor tissues compared to those in control tissues.

**Conclusions:**

The TIIC-based TMEscore was associated with the survival of CRC patients who underwent fluoropyrimidine-based chemotherapy, and predicted the response to chemotherapy. The TMEscore-related gene signature had a better predictive value for response to chemotherapy than for survival.

## Introduction

The prognosis of patients with colorectal cancer (CRC) is strongly dependent on the clinical stage, as the 5-year survival rate of patients at an advanced stage is much lower than that of patients at an early stage (1). Unfortunately, most patients with CRC are initially diagnosed at an advanced stage. Chemotherapy remains an important and effective approach for the treatment of patients with CRC, particularly those at advanced stages. Fluoropyrimidine-based chemotherapy, including FOLFOX, FOLFIRI, or CapeOX, is recommended as the first-line treatment for patients with unresectable and/or recurrent CRC (2, 3). However, chemotherapy resistance, which occurs in a significant proportion of patients and results in tumor progression and ultimately death, is a major obstacle to the successful treatment of CRC. Many mechanisms have been found to underlie the occurrence of chemotherapy resistance, including alterations in drug metabolism, aberrant DNA repair and proliferation of cancer cells, activation of detoxifying enzymes, and cancer cell death inhibition (4, 5). In recent decades, growing evidence has shown that alteration of the tumor microenvironment (TME) is an important factor that significantly contributes to chemotherapy resistance (6).

The TME is composed of tumor cells, stromal cells, immune cells, and other cell types. Changes in the cell diversity and cytokines in the TME contribute to the pathogenesis, progression, therapy response, and prognosis of cancers (7, 8). Alterations in TME cells, especially tumor-infiltrating immune cells (TIICs), and the protein molecules and cytokines secreted by these cells, mediate the occurrence of chemotherapy resistance (9). Recently, the association between TIICs and chemotherapy resistance in CRC has been reported in several studies. For example, a study reported that the presence of T follicular helper cells and M0 macrophages in the TME of patients with CRC who underwent fluoropyrimidine-based chemotherapy were associated with better survival rates, whereas eosinophils in the TME were associated with worse survival rates (10). Another study used a TME-specific gene signature to identify CRC subtypes. In this study, a “signature associated with FOLFIRI resistance and the microenvironment” (SFM) was constructed to identify both TIICs and drug sensitivity in CRC patients (11). A single-cell atlas of liver metastases of CRC reveals tumors treated with preoperative chemotherapy show activation of B cells, lower diversity of tumor-associated macrophages with immature and less activated phenotype, lower abundance of both dysfunctional T cells and ECM-remodeling cancer-associated fibroblasts, and accumulation of myofibroblasts (12). These results demonstrate that TIICs in the TME could be used to identify patients with chemoresistance and predict the prognosis in CRC patients undergoing chemotherapy.

The TMEscore is an index that reflects the fraction of immune cells and is calculated from a gene expression matrix using principal component analysis (PCA) (13). Compared with the immune cell fraction, the TMEscore could be more reliable in representing the composition of the TME. The TMEscore has recently been used to predict the survival rate of patients with various cancer types, such as gastric cancer (13), glioma (14), and ovarian cancer (15). However, an association between the TMEscore and chemotherapy resistance has not been previously reported. Therefore, in this study, we calculated the TMEscore of patients with CRC who underwent fluoropyrimidine-based chemotherapy based on TIICs, and constructed a TMEscore-related gene signature to predict the survival rate of these patients. Our results provide novel insights into the role of TIIC-mediated chemotherapy resistance and precise treatment options for patients who undergo fluoropyrimidine-based chemotherapy.

## Materials and methods

### CRC dataset acquisition and preprocessing

The CRC datasets were retrieved and freely downloaded from the gene expression omnibus (GEO) database (www.ncbi.nlm.nih.gov/geo/). Datasets containing information from 2061 patients with CRC, including GSE72970 (n=124), GSE39582 (n=585), GSE87211 (n=363), GSE104645 (n=193), and GSE28702 (n=83) were included in the analysis. The cancer genome atlas (TCGA)-CORDREAD (n=635) dataset (transcripts per million normalized) with corresponding clinical information was downloaded freely from the Xena database (GDC hub: https://gdc.xenahubs.net). Four GEO datasets, GSE106582 (n=194), GSE31737 (n=80), GSE117606 (n=208), and GSE74602 (n=60), were downloaded to validate gene expression differences between tumor and control tissues. The GEO datasets were preprocessed by performing background adjustment using the RMA algorithm.

### Quantification of TIICs in the TME

The TIICs in the TME of CRC tissues were quantified using the CIBERSORT algorithm (16), which generates a fraction of 22 immune cell phenotypes by calculating gene expression. The CRC samples with CIBERSORT P of <0.05 were retained for subsequent analysis because this inferred a highly reliable cell composition.

### Consensus clustering analysis and differentially expressed gene analysis

The “ConsensusClusterPlus” package (17) was used to perform unsupervised consensus clustering of the samples based on the TIIC fraction. The cumulative distribution function (CDF) curves determined the optimal number of clusters, which was indexed by k values from 2 to 6. The DEG analyses between different clusters in each dataset were performed using the “limma” package. This package provides an integrated solution for analyzing data from gene expression experiments. It contains rich features for handling complex experimental designs. To date, the “limma” package is a classic method for the differentially expressed analysis of microarray datasets (18).

### Functional enrichment analysis for the DEGs

Gene set variation analysis (GSVA) was applied to screen significantly enriched pathways between the two clusters using the Molecular Signatures Database (MSigDB, version 7.4) (19). Those with a P value of <0.05 were considered significant pathway terms. Kyoto Encyclopedia of Genes and Genomes (KEGG) terms were identified with a cutoff P value of <0.01 and a false discovery rate of <0.05. These analyses were conducted using the clusterProfiler package. The clusterProfiler package provides a universal interface for functional enrichment analysis in thousands of organisms based on internally supported ontologies and pathways, as well as annotation data provided by users or derived from online databases (20).

### TMEscore calculation and cluster analysis

The TMEscore of the gene dataset was calculated using the “TMEscore” package (13, 21). This package provides functionality for calculating the TMEscore using PCA or z-score methods. For the dataset with survival data, this package divides the samples into high or low clusters based on the TMEscore and survival data.

### Chemotherapeutic drugs and immunotherapeutic response prediction

The potential chemotherapeutic drugs that were associated with the TMEscore clusters were screened using the “pRRophetic” package (22), which is used to calculate the half-maximal inhibitory concentration (IC50) based on the gene expression profile for 251 chemotherapeutic drugs. Furthermore, the response of the TMEscore clusters to immunotherapy was predicted by comparing the expression of immune checkpoint inhibitors.

### Screening and validation of key genes using multiple machine learning algorithms

The datasets were randomly classified into training and testing sets in a ratio of 7:3. First, support vector machine (SVM), decision tree (DT), and Extreme Gradient Boosting (XGBoost) algorithms were used to screen the most important genes *via* the e1071, rpart, and XGBoost packages in the R language. The predictive value of the three machine learning algorithms for important gene selection in the training set was estimated by the receiver operating characteristic (ROC) curves and areas under the curve (AUC) using the “pROC” package. Then, the intersecting genes among the three algorithms were considered as the key genes and visualized using a Venn diagram. Finally, multivariate logistic regression analysis was conducted to construct the predictive signature using the key genes, which evaluated the predictive value of the signature *via* AUC indices.

### Reverse transcription-polymerase chain reaction assay for clinical samples

The expression of key genes in the CRC clinical samples was determined using RT-PCR. Forty tumor tissues and their corresponding adjacent tissues were collected between February 2020 and August 2021. This study was approved by the hospital’s ethics committee. Total RNA extraction, RT-PCR, and analysis were performed as previously described (23). TaqMan probe-based RT-PCR was performed using a commercial kit (Thermo Fisher Scientific, Inc.) according to the manufacturer’s instruction. The primers used for RT-PCR were as follows: ADH1C forward: TGA TAA AGT CAT CCC GCT CTT T, reverse: CAT TCT CAT CCA CCA CTG TGT A; SLC26A2 forward: AAG AGC AAC ATA ACG TTT CAC C, reverse: GTC TGC ATT GAT CAT TGG TCT C; NANS forward: TCA GAA GCT CTT TCC TGA CAT T; reverse: GTC CAA AGT TAT GTG ACG TTC C. The reaction conditions for the RT-PCR were as follows: 1 cycle at 95°C for 5 min; 15 cycles at 95°C for 25 sec, 64°C for 20 sec, 72°C for 20 sec; and a final 31 cycles at 93°C for 25 sec, 64°C for 20 sec, 72°C for 20 sec. Amplicons were detected using capillary electrophoresis on an ABI 3130xl Genetic Analyzer (Life Technologies, Grand Island, NY).

### Statistical analysis

Data with normally distributed variables were tested using the unpaired Student’s *t* test for two-group comparisons; otherwise, the Mann–Whitney U-test was used. The Kruskal–Wallis test and one-way analysis of variance were used for multiple group comparisons. The chi-squared test was used to compare categorical variables. Kaplan–Meier and log-rank (Mantel–Cox) tests were employed to compare patient survival rates using the “survminer” package. All statistical analyses were conducted using the R language. P values of <0.05 were considered statistically significant. P values were two-sided.

## Results

### Establishment of clusters based on the TIIC fraction

**Figure 1:**
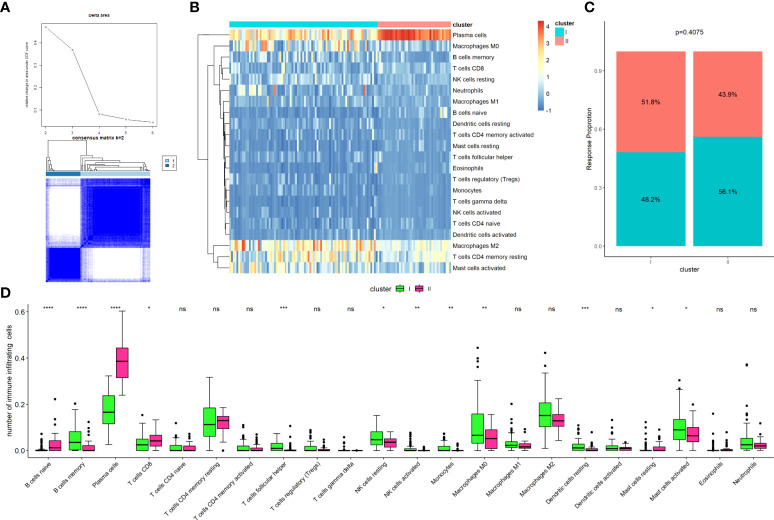
Establishment of clusters based on immune cells infiltrating fraction. **(A)** Unsupervised consensus clustering for the samples based on the TIICs infraction, with the k value as 2; **(B)** Clustering of the tumor-infiltrating immune cells; **(C)** Comparison of cluster frequency with the response status; **(D)** Comparison of tumor-infiltrating immune cells between Cluster I and Cluster II. *P < 0.05; **P < 0.01; ***P < 0.001; ns, not significant.

The GSE72970 dataset included 124 samples from CRC patients who underwent fluoropyrimidine-based chemotherapy, including FOLFOX, FOLFIRI, FOLFIRINOX, combined with bevacizumab. The dataset only included patients with metastatic CRC. Sixty-three patients had shown a response (complete response + partial response) to chemotherapy, whereas 61 patients had not (stable disease + progressive disease). The TIIC fraction of the dataset was calculated using the CIBERSORT algorithm. Then, the 22 TIIC fraction was clustered using unsupervised clustering methods *via* the ConsensusClusterPlus package. The results suggested that TIICs can be optimally divided into two clusters (cluster I, n=83; cluster II, n=41; **Figure 1A**). **Figure 1B** shows a heatmap of the clusters for the TIIC fraction. The frequency of the clusters was not significantly different to that of the response to chemotherapy (**Figure 1C**, P=0.407). **Figure 1D** shows a comparison of the TIIC fraction between clusters I and II. These results indicated that the TIICs of patients who underwent fluoropyrimidine-based chemotherapy could be divided into two clusters, and that the results were associated with the response to chemotherapy.

### GSVA and DEG analyses for the TIIC clusters

**Figure 2:**
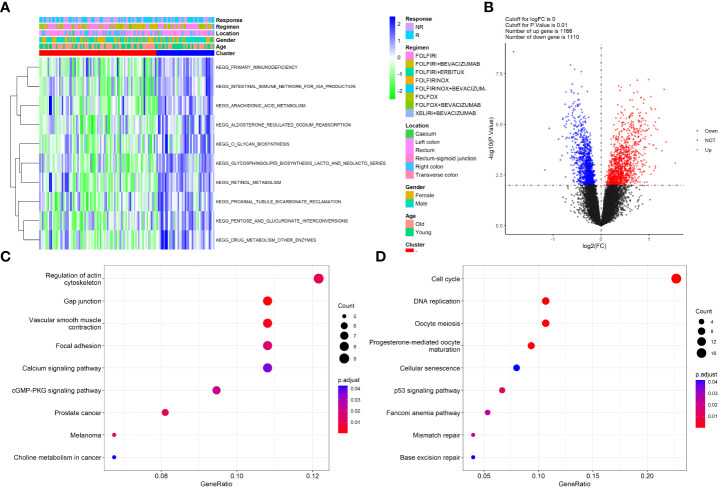
Functional enrichment of clusters and the DEGs. **(A)** Heatmap of GSVA analysis revealed the significant pathways that Clusters involved; **(B)** Volcano plot showed the DEGs between the two clusters; **(C)** KEGG pathways that up-regulated DEGs involved; **(D)** KEGG pathways that down-regulated DEGs involved.

To reveal the potential pathways involved in the TIIC clusters of patients with CRC, the GSVA algorithm was applied to clusters I and II. Generally, GSVA is used to estimate the variation in pathway activity over a sample population in an unsupervised manner. As illustrated in **Figure 2A**, the top three enriched pathways by GSVA between the two clusters were primary immunodeficiency, the intestinal immune network for IGA production, and arachidonic acid metabolism, suggesting that these two clusters were involved in the immune system processes. Next, DEGs between clusters I and II were screened using the “limma” package in the GSE72970 dataset with the criterion of a P value of <0.05. A total of 2267 immune cell-related DEGs were identified between the two clusters (**Figure 2B**). Afterwards, the pathways that the DEGs were involved in were determined using the top 200 upregulated and downregulated DEGs *via* the “clusterProfiler” package. The results revealed that the upregulated DEGs were mainly involved in the regulation of the actin cytoskeleton, calcium signaling pathway, and cGMP-PKG signaling pathway (**Figure 2C**); the downregulated DEGs were mainly involved in the cell cycle, DNA replication, and p53 signaling pathway (**Figure 2D**). Altogether, these results provided information on the role of TIIC clusters and DEGs in biological processes and the pathophysiology of diseases.

### TMEscore calculation and the association with chemotherapy response and survival

**Figure 3:**
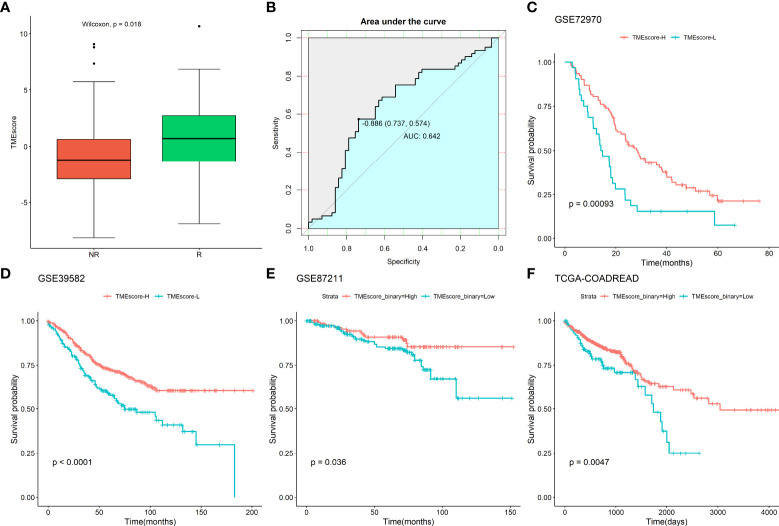
Association of TMEscore with chemotherapy response and survival in CRC patients. **(A)** Comparison of TMEscore between response and non-response to the chemotherapy; **(B)** ROC of the TMEscore in predicting the response to chemotherapy; Association of TMEscore with survival of CRC patients underwent chemotherapy in **(C)** GSE72970 dataset; **(D)** GSE39582 dataset; **(E)** GSE87211 dataset; **(F)** TCGA-CORDREAD dataset.

Next, the TMEscore was calculated by incorporating the 2267 DEGs and survival data of the GSE72970 dataset using the “TMEscore” package. The TMEscore was lower in those who did not respond to chemotherapy compared to those who did respond to chemotherapy (Wilcoxon test, P=0.018; **Figure 3A**). The ROC curve revealed that the TMEscore had a moderate predictive value for identifying patients who responded to chemotherapy (AUC:0.642, **Figure 3B**). Next, the samples were divided into two groups, TMEscore-High (TMEscore-H) and TMEscore-Low (TMEscore-L) based on the optimal cutoff point of the TMEscore. The Kaplan-Meier plot showed that there was a significantly different survival time between TMEscore-H and TMEscore-L patients (**Figure 3C**). Subsequently, the association of the TMEscore group with the survival of CRC patients who underwent fluoropyrimidine-based chemotherapy was validated in three datasets (GSE39582, n=585; GSE87211, n=363; TCGA- CORDREAD, n=635; **Figures 3C–F**). The results revealed that patients with a high TMEscore showed better survival time compared to those with a low TMEscore. In addition, we conducted subgroup analyses by dividing the samples into two groups according to the administration of irinotecan or oxaliplatin in combination with fluoropyrimidine. We then compared the TMEscore and other clinical parameters between the two groups, and the results failed to show any significance regarding the TMEscore, patients’ age, T stage, N stage, and response status (**Supplementary Table S1**). Altogether, these results indicate that the TMEscore based on the TIIC-related DEGs could predict the prognosis of CRC patients who underwent chemotherapy.

### Clinical therapeutic strategies using the TMEscore

**Figure 4:**
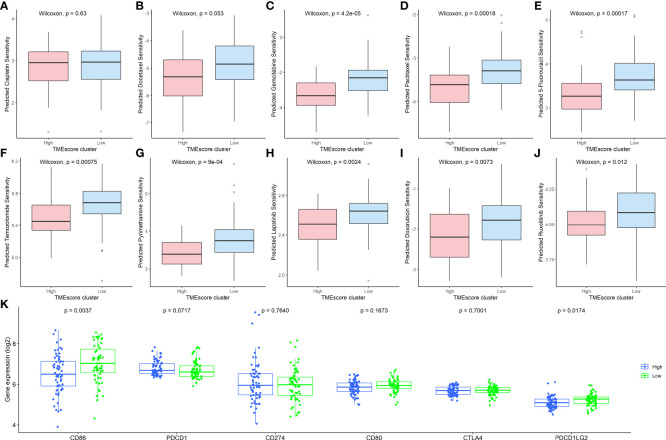
Clinical therapeutic strategies using the TIIC-related DEGs. Comparison of the IC50 values between the TMEscore-H and TMEscore-L groups of **(A)** Cisplatin; **(B)**: Docetaxel; **(C)** Gemcitabine; **(D)** Paclitaxel; **(E)** 5-Fluorouracil; **(F)** Temozolomide; **(G)** Pyrimethamine; **(H)** Lapatinib; **(I)** Doxorubicin; **(J)** Ruxolitinib; **(K)** Comparison of six immune checkpoint inhibitors between TMEscore clusters. TIIC, tumor-infiltrating immune cell; DEG, differentially expressed gene; IC50, half-maximal inhibitory concentration; TMEscore, tumor microenvironment score, TMEscore-H, high TMEscore; TMEscore-L, low TMEscore.

To explore the clinical therapeutic strategies using the TMEscore, we applied the “pRRophetic” package (22) to predict the potential therapeutic effects of CRC first-line chemotherapy drugs, including 5-fluorouracil, cisplatin, docetaxel, gemcitabine, and paclitaxel. As shown in **Figures 4A, B**, the IC50 of the TMEscore-H group was not significantly different to that of the TMEscore-L group for cisplatin and docetaxel (P>0.05), but was significantly different for 5-fluorouracil, gemcitabine, and paclitaxel (**Figures 4C–E**). Furthermore, five chemotherapy drugs (temozolomide (24), pyrimethamine (25), lapatinib (26), doxorubicin (27), and ruxolitinib (28)), which are reported to be associated with the treatment of CRC, showed significant differences between the TMEscore-H and TMEscore-L groups (**Figures 4F–J**). Thereafter, the expression levels of six known immune checkpoint inhibitors, namely PD1 (PDCD1), PD-L1, PD-L2 (PDCD1LG2), CD80, CD86, CTLA4, and CD274, were compared based on the TMEscore clusters. Only the expression of CD86 and PD-L2 was significantly different between the TMEscore-H and TMEscore-L groups (**Figure 4K**). In summary, these results suggest that CRC patients in the TMEscore-H group tend to benefit from specific chemotherapy drugs but are unlikely to benefit from immunotherapy.

### Machine learning methods screened key genes for chemotherapy response

**Figure 5:**
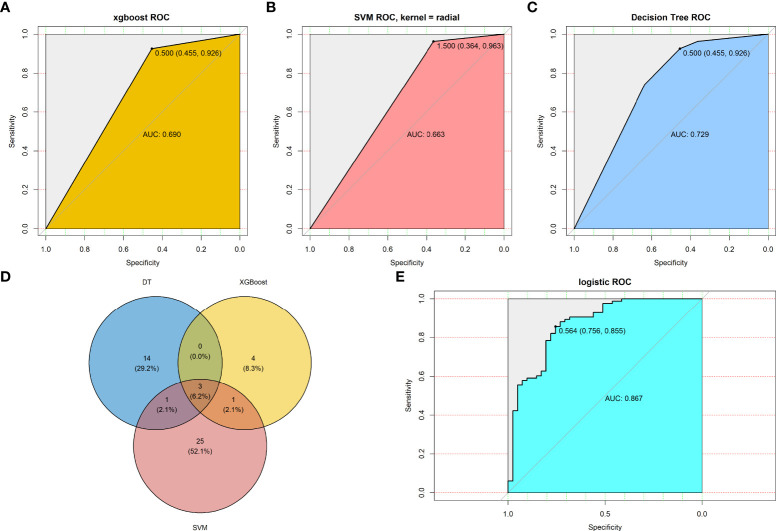
Machine learning methods screen key genes response to chemotherapy. Predictive value of TMEscore-relate genes in testing set by **(A)** XGBoost algorithms; **(B)** SVM algorithms; **(C)** DT algorithms; **(D)** Venn plot revealed three overlapped among the three machine learning methods; **(E)** Predictive value of the three TMEscore-relate genes signature in the response to chemotherapy by multivariate logistic regression.

To screen key genes associated with the response to chemotherapy of CRC patients, three machine learning methods (XGBoost, SVM, and DT) were employed to analyze the 2267 TIIC-related DEGs in the datasets. The samples were divided into training and test sets at a ratio of 7:3, with 768 and 321 samples in the training and test sets, respectively. The number of key genes screened by each method was 18, 30, and 8 in the training sets, which suggested that these genes are important in identifying the patients who respond to chemotherapy. Thereafter, we determined the predictive value of the above key genes using each machine learning method in the tested set. The results showed that the AUC values for the tested set were 0.690, 0.663, and 0.729, respectively (**Figures 5A–C**), suggesting that the key genes from each machine learning method achieved a moderate predictive value. Finally, three key genes (ADH1C, SLC26A2, and NANS) were obtained by overlapping the genes found using the three machine learning methods (**Figure 5D**). A logistic regression model was employed to determine the predictive value using these three key genes, and the results indicated that the signature constructed by the three key genes had good predictive performance, with an AUC of 0.867 (**Figure 5E**), indicating that the three key genes derived from the three machine learning methods improved the predictive performance of the model.

### Validation of the predictive value of the three-gene signature for chemotherapy response

**Figure 6:**
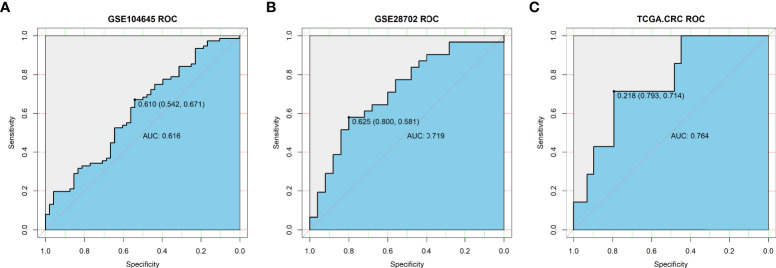
Validation of predictive value of TMEscore-relate genes signature in the response to chemotherapy in **(A)** GSE104645 dataset; **(B)** GSE28702 dataset; **(C)** TCGA-COADREAD dataset (36 patients underwent 5- Fluorouracil treatment).

To validate the predictive value of the three-gene (ADH1C, SLC26A2, and NANS) signature in patients with CRC who underwent fluoropyrimidine-based chemotherapy, two GEO datasets (GSE104645, n=193; GSE28702, n=83) were screened, which provided the response status data of CRC patients who underwent FOLFOX or FOLFOX-based regimens. Logistic regression was then employed to determine the predictive value of the three-gene signature. As **Figures 6A, B** shows, the predictive value of the signature constructed using the three genes was moderate, with AUC values of 0.616 and 0.719, respectively. Next, samples from patients who only underwent 5- fluorouracil treatment (n=36) were extracted from the TCGA-COADREAD dataset, and the predictive value increased, with an AUC value of 0.764 (**Figure 6C**). These results indicate that the signature constructed by ADH1C, SLC26A2, and NANS could help to identify patients who will probably benefit from fluoropyrimidine-based chemotherapy.

### Validation of the expression of key genes in external cohorts and clinical samples

**Figure 7:**
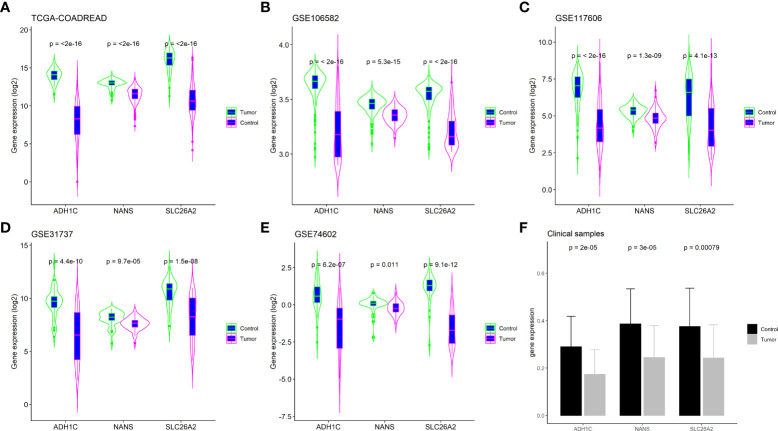
Validation key genes expression in external cohorts and clinical samples. **(A)** TCGA-COADREAD dataset; **(B)** GSE106582 dataset; **(C)** GSE117606 dataset; **(D)** GSE31737 dataset; **(E)** GSE74602 dataset; **(F)** Clinical samples by RT-PCR assay.

The expression of ADH1C, SLC26A2, and NANS was determined in the TCGA-COADREAD dataset (n=689) and four large external cohorts (GSE106582, n=194; GSE31737, n=80; GSE117606, n=208; GSE74602, n=60), and the expression of the three genes were compared between tumor and control tissues. As shown in **Figures 7A–E**, the results from the above five datasets revealed that the levels of all three genes were decreased in tumor tissues compared with those in control tissues in CRC samples (P<0.01). Finally, clinical samples of CRC were collected and gene expression was tested using RT-PCR. Consistent with the results from the datasets, the expression levels of ADH1C, SLC26A2, and NANS were substantially decreased in tumor tissues compared with those in the corresponding adjacent tissues (P<0.01; **Figure 7F**). These results demonstrated that the levels of ADH1C, SLC26A2, and NANS were decreased in CRC tissues compared to those in control tissues.

## Discussion

Currently, fluoropyrimidine-based chemotherapy regimens, mainly 5-fluorouracil and its oral prodrug capecitabine combined with irinotecan or oxaliplatin, remain the mainstay of treatment for many types of solid tumors (29), such as CRC, gastric cancer, and pancreatic cancer. However, only half of the patients respond to this regimen, which substantially hampers the treatment effect (30). Due to the relatively slow advancement of new agents to substitute traditional chemotherapeutics, predicting whether the patient will develop fluoropyrimidine-based chemotherapy resistance is critical in achieving effective clinical management of patients with CRC. Many biomarkers have been shown to be associated with fluoropyrimidine-based chemotherapy resistance, such as complement component 3 (31), ZEB2 (32), and PKM2 (33). These biomarkers may help identify patients who are suitable for fluoropyrimidine-based chemotherapy.

In the present study, the TIIC fraction was analyzed in a dataset with information on fluoropyrimidine-based chemotherapy. It was found that TIICs could be divided into two clusters, which were associated with the response to chemotherapy. The DEGs between the two clusters of TIICs were screened and then used to calculate the TMEscore and construct the TMEscore signature. These results indicated that the TMEscore was significantly associated with the survival of patients with CRC who underwent chemotherapy, suggesting that it could serve as an important indicator for predicting the prognosis of patients with CRC. Given that chemotherapy resistance is induced not only by a variety of cells, but also by protein molecules and cytokines in the cells, the genes that represent the TMEscore signature were also screened using three machine learning methods; three genes with good performance in predicting the response to chemotherapy in CRC patients were identified. Finally, the expression of these three genes was validated in larger cohorts and clinical samples. These results demonstrated that TIICs were associated with the response to fluoropyrimidine-based chemotherapy in patients with CRC, and that the TMEscore signature derived from TIICs could predict patient survival rate. In addition, the genes representative of the TMEscore signature could better predict the response to chemotherapy, rather than patient survival rates.

Previous studies have shown that TIICs are associated with chemotherapy resistance. The cytokine IL-22, which is produced by T and natural killer cells, protects CRC cells from chemotherapy by activating the STAT3 pathway and inducing the autocrine expression of IL-8 (34). A study described that 5-fluorouracil-based chemotherapy regimens increase the expression of CXCR2 and ligand CXCL7 in liver metastasis of CRC, thus explaining the aggressiveness of relapsed drug-resistant tumors (35). The TMEscore, a novel index that characterizes TIICs, is associated with the survival of patients with several cancer types. Since the TMEscore is calculated by the DEGs from different clusters, the TMEscore-based signature generally has a more reliable predictive value compared to that of individual TIIC fractions. Studies have also reported that the TMEscore could predict the responses to several immunotherapies in neuroblastoma (36) and breast cancer (37).

Although the TMEscore is closely associated with the response to chemotherapy or immunotherapy, the clinical application value of the TMEscore remains to be determined. In this study, by overlapping the results from the three machine learning methods, three genes (ADH1C, SLC26A2, and NANS) were identified that showed good predictive value regarding the response to chemotherapy. ADH1C has been shown to inhibit the progression of CRC through the ADH1C/PHGDH/PSAT1/serine metabolic pathway (38), and downregulation of ADH1C has been associated with poor prognosis in patients with CRC (39). Higher SLC26A2 expression in tumor tissues indicates a longer survival for patients with CRC (40). NANS, together with seven other genes, could better predict the survival rate of patients with CRC (41). The results also suggested that these three genes participate in the development of CRC, and can be used to predict the prognosis of CRC patients. However, the role of these three genes in chemotherapy resistance have previously not been investigated. Thus, future relevant studies may further uncover the mechanism of chemotherapy resistance of each gene, and their association with the immune cells in CRC is worth further exploration.

Compared to previous studies (42–44), this study identified the prognostic value of the TMEscore in CRC and the predictive value of TMEscore-related genes in the response to fluoropyrimidine-based chemotherapy, which has not previously been reported. Moreover, our results were derived from a gene dataset with large cohorts of public databases, and validated in several independent cohorts, thereby guaranteeing the robustness of the results. Furthermore, the results from the individual analysis of each gene were consistent with the overall analysis, which makes the utility of our findings in clinical decision-making easier, and doctors can estimate the prognosis of CRC patients based on fewer gene expressions, and administer appropriate treatment.

However, there were several limitations in this study. Firstly, the GSE72970 dataset used in the analysis only includes patients with metastatic CRC who receiving fluoropyrimidine-based chemotherapy as first-line therapy; therefore, the results might not be representative of non-metastatic CRC. Secondly, the fraction of TIICs obtained were from microarray datasets, and the exact number of TIICs will still need to be determined by the immunohistochemistry method or flow cytometry. Thirdly, the predictive value of the TMEscore in this study was verified using microarray datasets; thus, the exact predictive value will still need to be validated in a larger clinical cohort. Finally, more experiments are needed to confirm the association of the key genes with resistance to chemotherapy agents.

## Conclusion

In the present study, the TIIC-based TMEscore was associated with the survival rate of patients with CRC who underwent fluoropyrimidine-based chemotherapy and could predict the response to chemotherapy. Furthermore, the TMEscore-related gene signature had a better predictive value for the response to chemotherapy compared to that of survival rates.

## Data availability statement

The original contributions presented in the study are included in the article/**Supplementary Material**. Further inquiries can be directed to the corresponding authors.

## Ethics statement

This study was reviewed and approved by Ethics Committee of Guangxi Medical University Cancer Hospital. The patients/participants provided their written informed consent to participate in this study.

## Author contributions

Study concept and design: X-WG, ZD and RL. Collection and assembly of data: X-WG and B-LH. Performed the experiment: S-QL, B-LH and X-WG. Data analysis and interpretation: S-QL, B-LH and X-WG. Manuscript writing and review: All authors. All authors contributed to the article and approved the submitted version.

## Funding

This study was partially supported by research funding from the National Natural Science Foundation of China (No. 82060104; No. 81860417; No. 82160446).

## Acknowledgments

We thank Dr. Jianming Zeng (University of Macau), and all the members of his bioinformatics team, biotrainee, for generously sharing their experience and codes.

## Conflict of interest

The authors declare that the research was conducted in the absence of any commercial or financial relationships that could be construed as a potential conflict of interest.

## Publisher’s note

All claims expressed in this article are solely those of the authors and do not necessarily represent those of their affiliated organizations, or those of the publisher, the editors and the reviewers. Any product that may be evaluated in this article, or claim that may be made by its manufacturer, is not guaranteed or endorsed by the publisher.
