# Supplementary material for: Tumor-infiltrating immune cells based TMEscore and related gene signature is associated with the survival of CRC patients and response to fluoropyrimidine-based chemotherapy
Source: Front Oncol. 2022 Aug 30;12:953321. doi: 10.3389/fonc.2022.953321 (PMC9468757; doi:10.3389/fonc.2022.953321)
Supplement: Supplementary file 1 [file Table_1.docx]

**Table S1 Comparison of fluoropyrimidine with oxaliplatin and irinotecan**

|  | FU+ irinotecan(N=91) | FU+ OXA(N=32) | P value |
| --- | --- | --- | --- |
| Age | 61.0±11.3 | 63.6±11.8 | 0.272 |
| Tumor location |  |  | 0.478 |
| Caecum | 2 (2.20%) | 1 (3.12%) |  |
| Left colon | 35 (38.5%) | 15 (46.9%) |  |
| Rectum | 11 (12.1%) | 7 (21.9%) |  |
| Rectum-sigmoid junction | 16 (17.6%) | 3 (9.38%) |  |
| Right colon | 25 (27.5%) | 6 (18.8%) |  |
| Transverse colon | 2 (2.20%) | 0 (0.00%) |  |
| T stage |  |  | 0.532 |
| pT1 | 1 (1.10%) | 0 (0.00%) |  |
| pT2 | 5 (5.49%) | 2 (6.25%) |  |
| pT3 | 38 (41.8%) | 11 (34.4%) |  |
| pT4 | 29 (31.9%) | 8 (25.0%) |  |
| pTX | 18 (19.8%) | 11 (34.4%) |  |
| N stage |  |  | 0.403 |
| pN0 | 11 (12.1%) | 3 (9.38%) |  |
| pN1 | 20 (22.0%) | 7 (21.9%) |  |
| pN2 | 42 (46.2%) | 11 (34.4%) |  |
| pNX | 18 (19.8%) | 11 (34.4%) |  |
| Response status |  |  | 0.166 |
| Non-response | 49 (53.8%) | 12 (37.5%) |  |
| Response | 42 (46.2%) | 20 (62.5%) |  |
| TMEscore | -0.24±3.70 | 0.59±3.31 | 0.242 |

FU: fluoropyrimidine; OXA: oxaliplatin
